# Supplementary material for: Impacts of National Drug Price Negotiation on Expenditure, Volume, and Availability of Targeted Anti-Cancer Drugs in China: An Interrupted Time Series Analysis
Source: Int J Environ Res Public Health. 2022 Apr 11;19(8):4578. doi: 10.3390/ijerph19084578 (PMC9025142; doi:10.3390/ijerph19084578)
Supplement: Supplementary file 1 [file ijerph-19-04578-s001.zip › ijerph-1611537-supplementary.pdf]

Supplementary materials

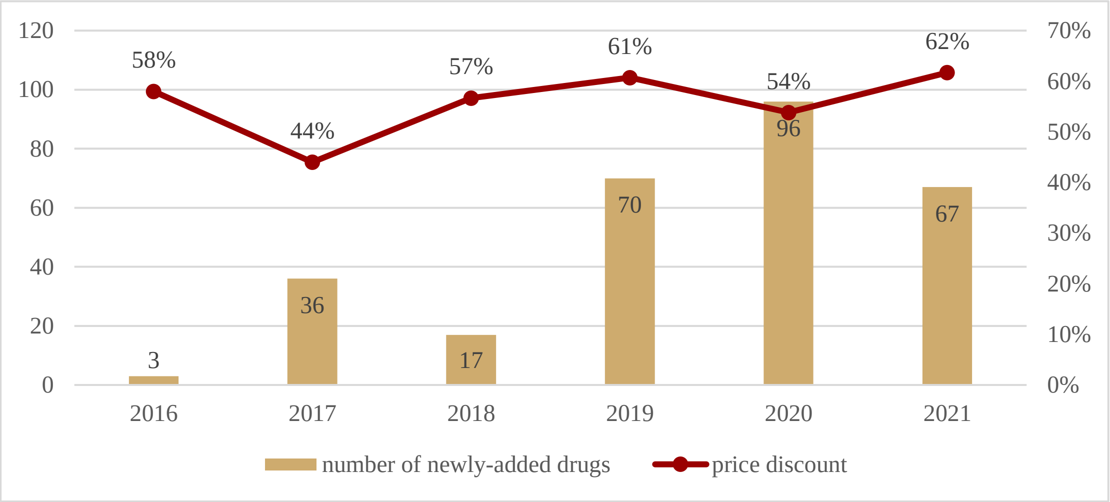

Figure S1. Number of newly-added drugs and their average price discount in six rounds of national drug price negotiation in China

A. Expenditure

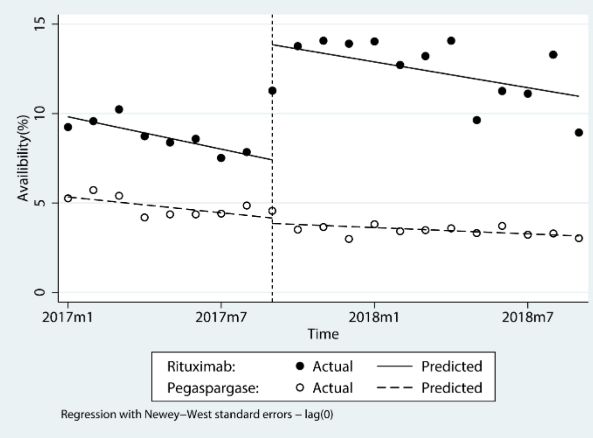

(a) Rituximab

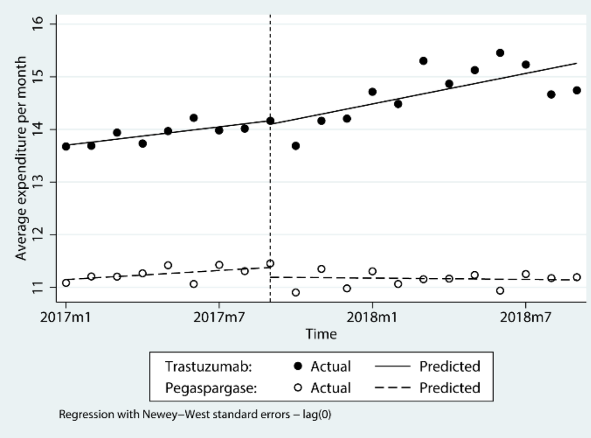

(b) Trastuzumab

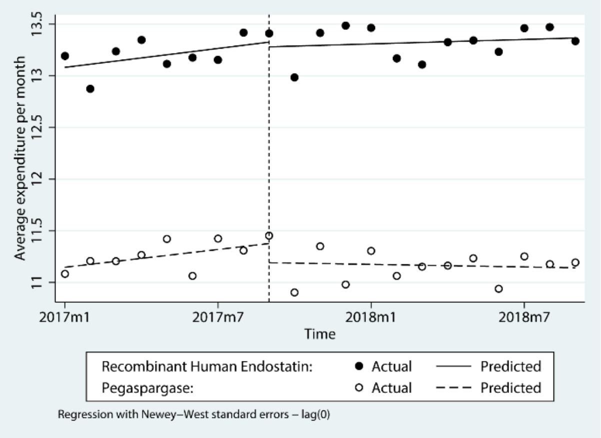

(c) Recombinant Human Endostatin

B. Volume

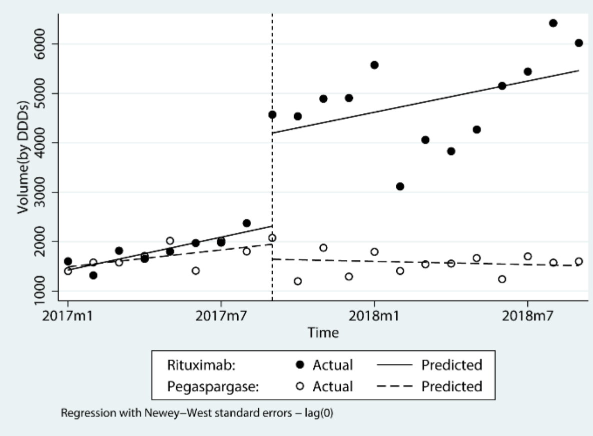

(a) Rituximab

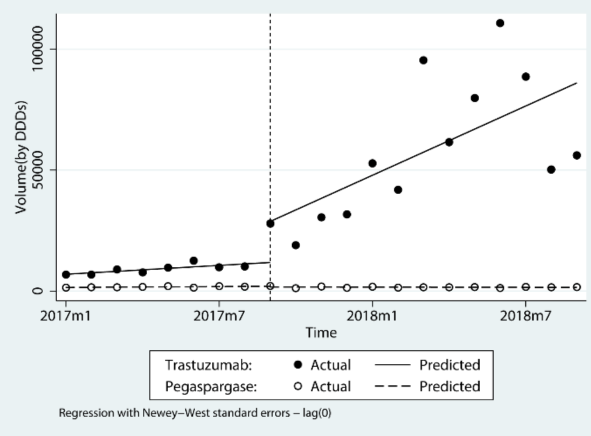

(b) Trastuzumab

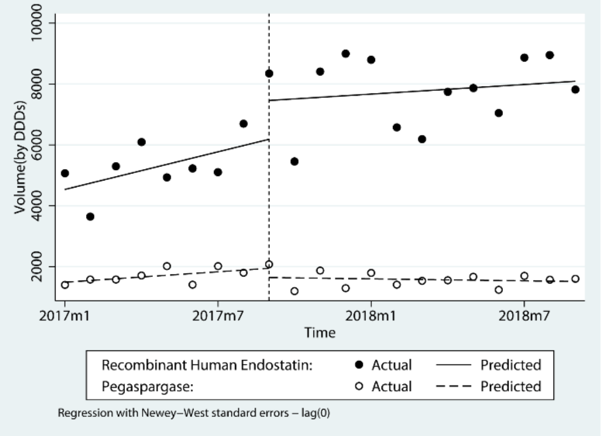

(c) Recombinant Human Endostatin

C. Availability

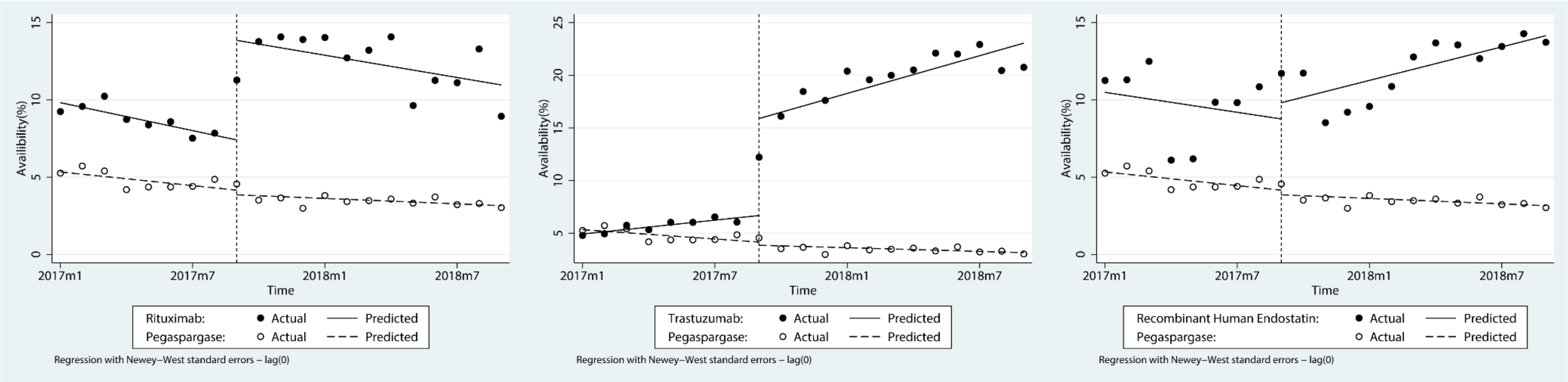

(a) Rituximab (b) Trastuzumab (c) Recombinant Human Endostatin

Figure S2. ITS analysis for average procurement expenditure, volume and availability in eastern provinces. A: average expenditure; B: average procurement volume (by DDDs); C: availability. (**a**) Rituximab; (**b**) Trastuzumab; (**c**) Recombinant human endostatin.

A. Expenditure

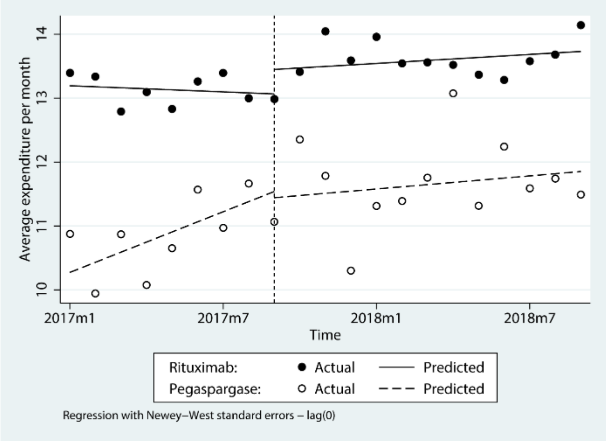

(a) Rituximab

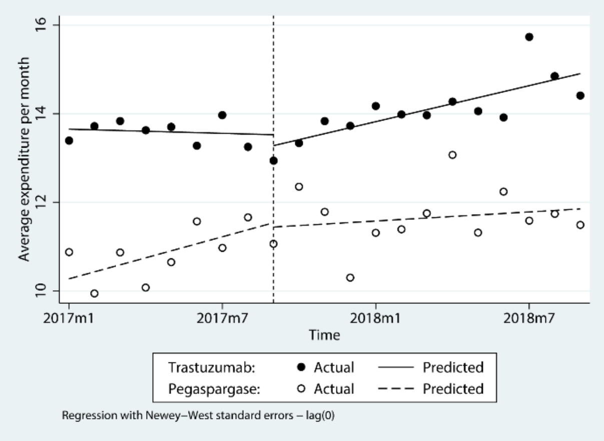

(b) Trastuzumab

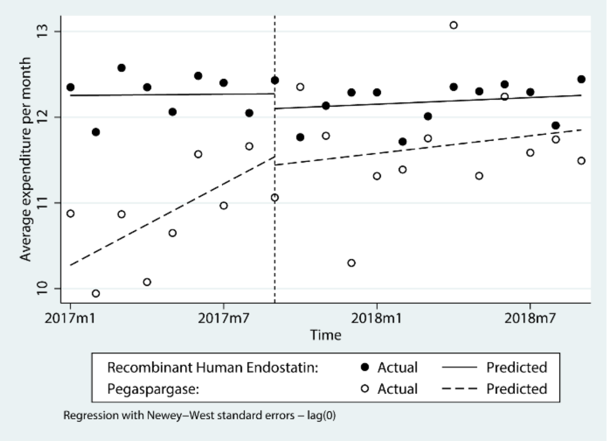

(c) Recombinant Human Endostatin

B. Volume

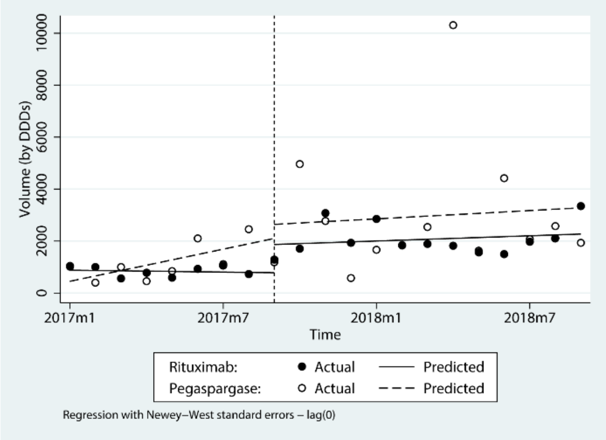

(a) Rituximab

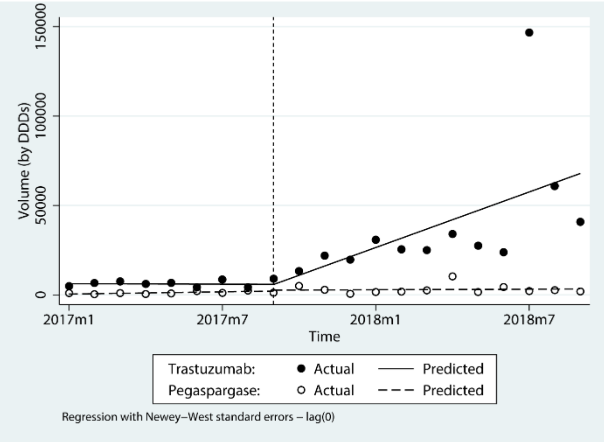

(b) Trastuzumab

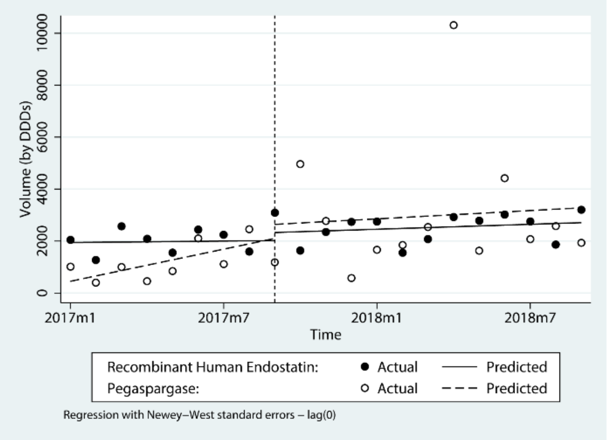

(c) Recombinant Human Endostatin

C. Availability

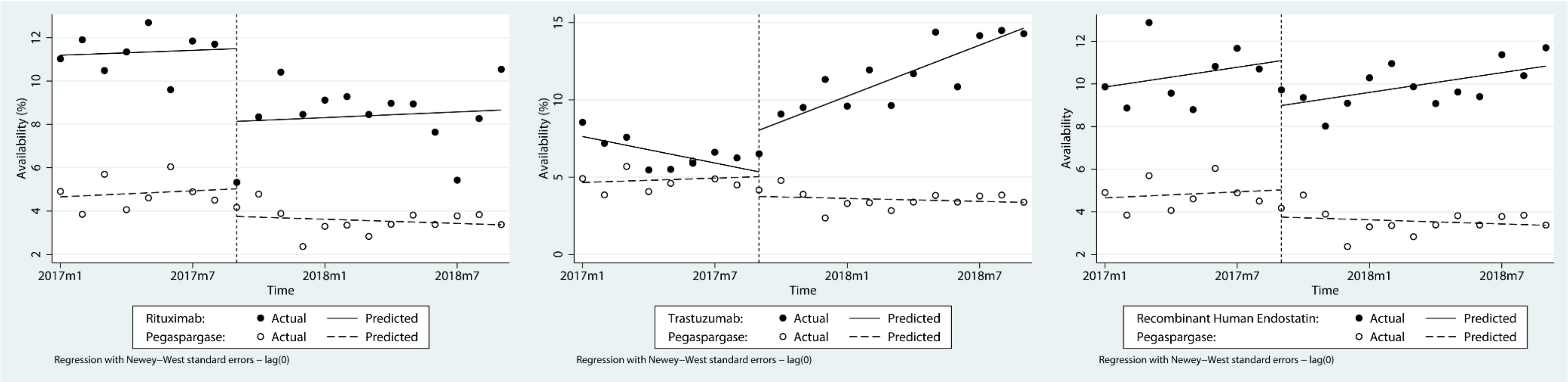

(a) Rituximab (b) Trastuzumab (c) Recombinant Human Endostatin

Figure S3. ITS analysis for average procurement expenditure, volume and availability in middle provinces. A: average expenditure; B: average procurement volume (by DDDs); C: availability. (**a**) Rituximab; (**b**) Trastuzumab; (**c**) Recombinant human endostatin.

A. Expenditure

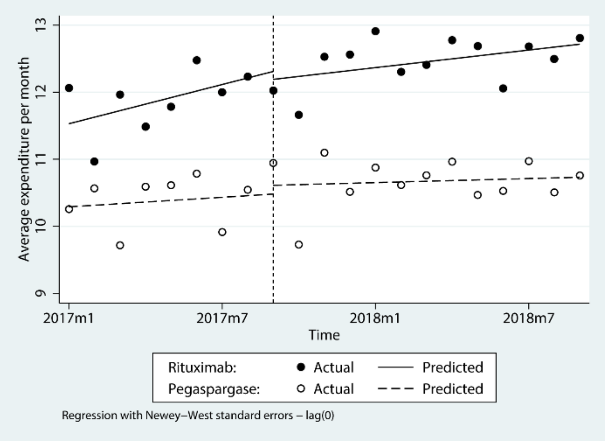

(a) Rituximab

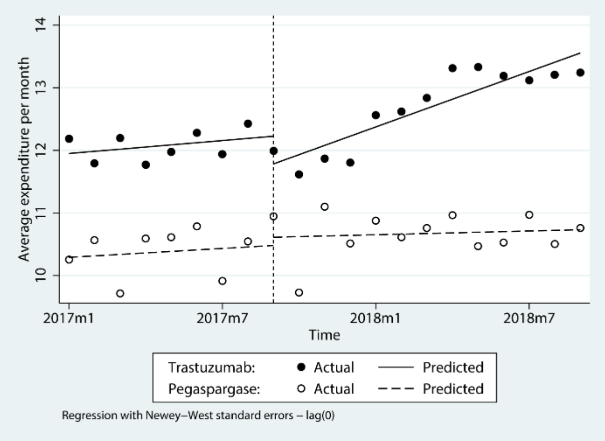

(b) Trastuzumab

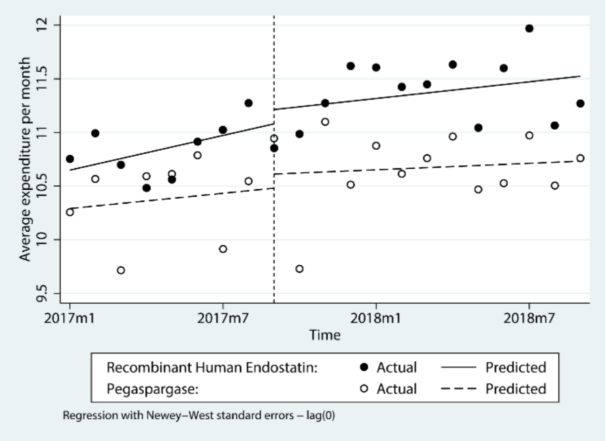

(c) Recombinant Human Endostatin

B. Volume

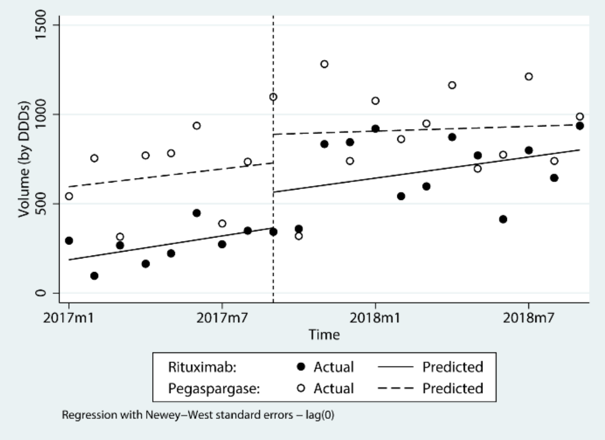

(a) Rituximab

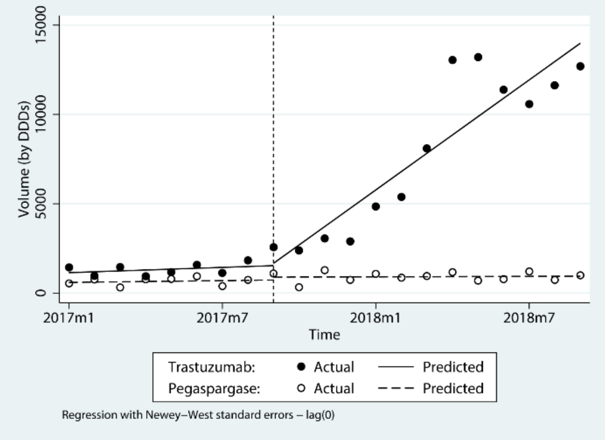

(b) Trastuzumab

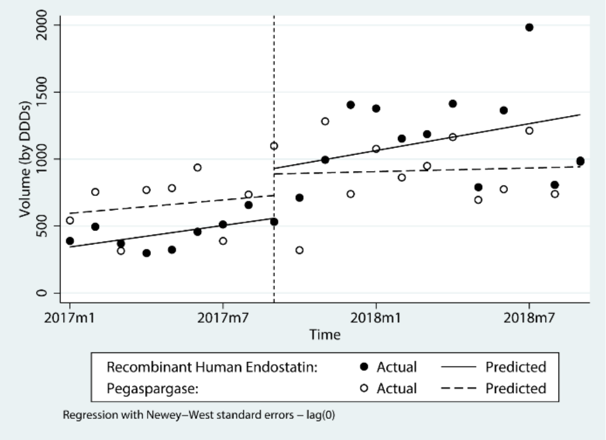

(c) Recombinant Human Endostatin

C. Availability

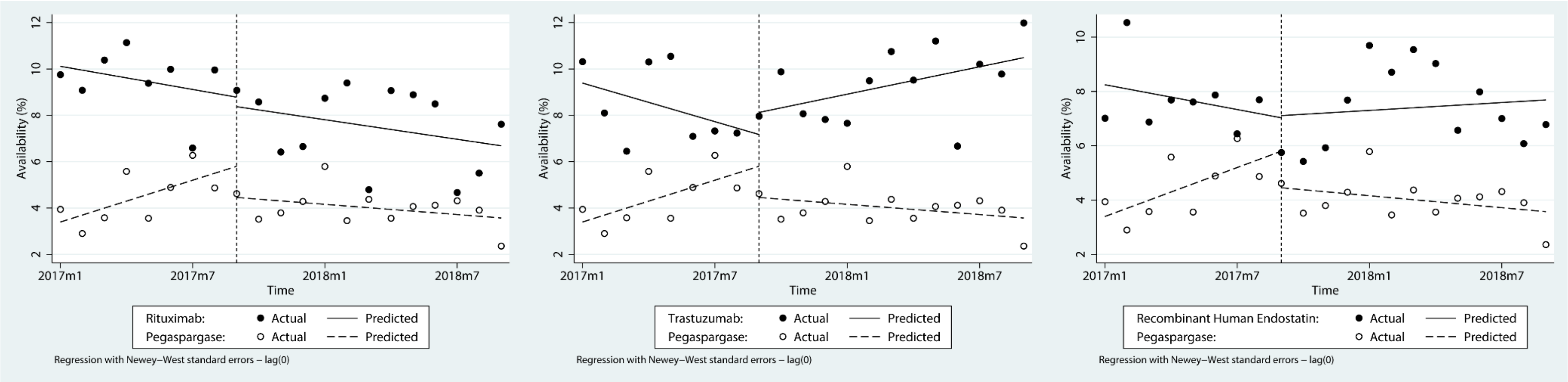

(a) Rituximab (b) Trastuzumab (c) Recombinant Human Endostatin

Figure S4. ITS analysis for average procurement expenditure, volume and availability in western provinces. A: average expenditure; B: average procurement volume (by DDDs); C: availability. (**a**) Rituximab; (**b**) Trastuzumab; (**c**) Recombinant human endostatin.

Table S1. Changes in level and trend of expenditure, volume and availability across eastern, middle and western provinces

Molecule	Variables	Average expenditure（log）			Volume (DDDs)			Availability (%)		
		East	Middle	West	East	Middle	West	East	Middle	West
Rituximab	Baseline difference(β4)	2.51***	2.92***	1.24***	-65.77	428.44	-408.83***	4.56***	6.73***	6.89***
		(0.09)	(0.40)	(0.42)	(142.56)	(350.19)	(130.70)	(0.40)	(0.58)	(0.91)
	Baseline trend difference(β5)	0.02	-0.17**	0.07	53.95	-218.48**	5.63	-0.21**	-0.08	-0.55**
		(0.02)	(0.08)	(0.08)	(36.87)	(93.18)	(31.54)	(0.08)	(0.14)	(0.25)
	Difference in level change (β6)	0.25	0.48	-0.25	2,189.33***	545.38	39.73	5.58***	-1.79	1.36
		(0.19)	(0.56)	(0.50)	(450.80)	(1,175.59)	(290.82)	(0.76)	(1.43)	(1.56)
	Difference in trend change (β7)	0.01	0.16	-0.04	62.10	198.85	9.62	0.22	0.13	0.71**
		(0.03)	(0.10)	(0.09)	(65.00)	(166.84)	(42.66)	(0.16)	(0.22)	(0.26)
Trastuzumab	Baseline difference(β4)	2.55***	3.38***	1.66***	5,432.62***	5,784.72***	545.22**	-0.41	2.98***	5.99***
		(0.07)	(0.40)	(0.26)	(450.54)	(921.53)	(226.21)	(0.33)	(0.71)	(1.08)
	Baseline trend difference(β5)	0.03	-0.17*	0.01	555.59***	-246.66	32.56	0.37***	-0.33**	-0.58***
		(0.02)	(0.09)	(0.06)	(158.68)	(293.18)	(58.23)	(0.08)	(0.15)	(0.20)
	Difference in level change (β6)	0.12	-0.15	-0.57	17,257.61**	-679.70	-28.81	9.51***	3.97***	1.05
		(0.26)	(0.59)	(0.46)	(8,044.22)	(7,261.41)	(723.31)	(1.38)	(1.07)	(1.47)
	Difference in trend change (β7)	0.07*	0.28**	0.13*	4,228.73**	5,371.25**	989.78***	0.29	0.92***	0.89***

		(0.04)	(0.11)	(0.07)	(1,744.49)	(2,491.55)	(118.96)	(0.20)	(0.18)	(0.26)
	Baseline difference(β4)	1.93***	1.98***	0.36	3,046.65***	1,491.51***	-251.22*	6.19***	5.20***	4.85***
		(0.12)	(0.42)	(0.25)	(538.97)	(457.11)	(127.62)	(0.53)	(1.00)	(1.23)
	Baseline trend difference(β5)	0.00	-0.16*	0.03	148.95	-196.68*	10.14	-0.07	0.11	-0.45*
		(0.03)	(0.09)	(0.06)	(122.96)	(112.40)	(31.91)	(0.13)	(0.18)	(0.25)
RHE	Difference in level change (β6)	0.14	-0.08	0.00	1,576.99	-231.30	211.14	0.62	-0.83	1.32
		(0.22)	(0.55)	(0.46)	(979.42)	(1,202.51)	(323.61)	(1.22)	(0.97)	(1.35)
	Difference in trend change (β7)	0.01	0.13	-0.01	-85.66	175.16	18.88	0.45**	0.08	0.62**
		(0.03)	(0.10)	(0.07)	(153.54)	(176.03)	(51.53)	(0.17)	(0.19)	(0.28)

Note: Standard errors in parentheses, (*** p<0.01, ** p<0.05, * p<0.1). Eastern provinces: Beijing, Hebei, Zhejiang and Fujian; Middle provinces: Anhui, Henan, Jilin and Hubei; Western provinces: Shaanxi, Chongqing and Guangxi.
